# Inoculated Cell Density as a Determinant Factor of the Growth Dynamics and Metastatic Efficiency of a Breast Cancer Murine Model

**DOI:** 10.1371/journal.pone.0165817

**Published:** 2016-11-07

**Authors:** Ana C. Gregório, Nuno A. Fonseca, Vera Moura, Manuela Lacerda, Paulo Figueiredo, Sérgio Simões, Sérgio Dias, João Nuno Moreira

**Affiliations:** 1 CNC—Center for Neurosciences and Cell Biology, University of Coimbra, Coimbra, Portugal; 2 IIIUC–Institute for Interdisciplinary Research, University of Coimbra, Coimbra, Portugal; 3 FFUC—Faculty of Pharmacy, Pólo das Ciências da Saúde, University of Coimbra, Coimbra, Portugal; 4 TREAT U, SA, Coimbra, Portugal; 5 IPATIMUP–Institute of Molecular Pathology and Immunology, University of Porto, Porto, Portugal; 6 IPOFG-EPE–Portuguese Institute of Oncology Francisco Gentil, Coimbra, Portugal; 7 IMM–Institute of Molecular Medicine, Faculty of Medicine, University of Lisbon, Lisbon, Portugal; University of Texas Health Science Center, UNITED STATES

## Abstract

4T1 metastatic breast cancer model have been widely used to study stage IV human breast cancer. However, the frequent inoculation of a large number of cells, gives rise to fast growing tumors, as well as to a surprisingly low metastatic take rate. The present work aimed at establishing the conditions enabling high metastatic take rate of the triple-negative murine 4T1 syngeneic breast cancer model. An 87% 4T1 tumor incidence was observed when as few as 500 cancer cells were implanted. 4T1 cancer cells colonized primarily the lungs with 100% efficiency, and distant lesions were also commonly identified in the mesentery and pancreas. The drastic reduction of the number of inoculated cells resulted in increased tumor doubling times and decreased specific growth rates, following a Gompertzian tumor expansion. The established conditions for the 4T1 mouse model were further validated in a therapeutic study with peguilated liposomal doxorubicin, in clinical used in the setting of metastatic breast cancer. Inoculated cell density was proven to be a key methodological aspect towards the reproducible development of macrometastases in the 4T1 mouse model and a more reliable pre-clinical assessment of antimetastatic therapies.

## Introduction

The manifestation of metastases is predictive of poor clinical outcome [1–4], and prevails one of the most challenging issues faced by cancer treatment today. A continuous effort in dissecting the biological processes behind cancer cell dissemination has been pushing forward our understanding of the disease and uncovering vulnerabilities that may be exploited for the development of novel agents to treat metastatic cancer.

Mouse models are crucial to our comprehensive knowledge on the molecular basis and pathogenesis of cancer disease [5]. Nevertheless, a major impediment for the study of metastases has been the unavailability of suitable mouse models that accurately recapitulate the complexity of human tumor progression [6, 7]. To better mimic the development of metastases in humans, several parameters need to be considered in a mouse model, namely location and implantation method of the primary tumor, interaction of cancer cells with the microenvironment at the primary and secondary sites, dissemination routes and time-to-progression of the disease. Subcutaneous transplantation of human (xenograft) and murine (allograft) cell lines into mice, and genetic engineered mice, are widely used for the establishment of pre-clinical models [6, 8]. In the subcutaneous model, ectopic location of cancer cells usually fails to produce metastases, owing to the limited tumor microenvironment generated [9]. Furthermore, surgical resection of primary tumors is often imperative in order to prolong mice survival and enable the development of spontaneous metastases [6]. Genetic engineered mouse models surpass some of these constrains, offering the possibility of orthotopic neoplastic generation in immune competent hosts [6, 8]. Nevertheless, metastatic lesions may appear only upon long latency periods and generally their incidence is low [6, 8]. Even though the existing pre-clinical models still offer valuable information about the biology, molecular basis and therapeutic opportunities, the setting up of spontaneous metastases faces several challenges, and improvement of its modeling remains of major importance [6, 7, 10].

The murine 4T1 breast carcinoma cell line has remarkable tumorigenic and invasive characteristics. Upon injection in the mammary gland of BALB/c mice, 4T1 cells spontaneously generate tumors and are described to metastasize to the lungs, liver, lymph nodes, brain and bones, in a way that closely resembles human breast cancer [11]. Owing to its characteristics, 4T1 cells have been widely used to study stage IV human breast cancer [12–15]. Moreover, 4T1 murine tumors represent a clinically relevant triple-negative breast cancer model [16–18], which, alongside the cancer cell invasion and metastization, is an important challenge due to its lack of responsiveness to endocrine therapy. However, 4T1 metastatic breast cancer model suffers from the liability of fast growing tumors enhanced by the frequent inoculation of a large number of cells, rendering a tumor microenvironment that does not recapitulate human breast tumors, early mice euthanasia [15, 19–25], along with a surprisingly low metastatic take rate.

Notwithstanding the widespread use of the 4T1 animal model, some of the aforementioned issues truly limit its usefulness to understand the biology of metastatic breast cancer and therefore the identification of novel therapeutic opportunities and the corresponding proof of concept. The need of translatable and predictive tumor models is a recognized need for successful drug development. The present work aimed at establishing the conditions enabling high metastatic take rate of the widespread triple-negative murine 4T1 syngeneic breast cancer model, towards a more reliable pre-clinical screening of anticancer drugs. It was demonstrated that the significant reduction of 4T1 cancer cell density implanted orthotopically, is a key methodological aspect underlying the reproducible development of macrometastases in this mouse model.

## Materials and Methods

### Ethics statement

All animal experiments were conducted according to human standards of animal care (2010/63/EU directive and Portuguese Act 113/2013, for the use of experimental animals), and approved by the corresponding national authority (Direção Geral de Alimentação e Veterinária). Animals were euthanized by cervical dislocation.

### Materials

Ethylenediaminetetraacetic acid disodium salt dihydrate, potassium phosphate monobasic, disodium phosphate anhydrous, potassium chloride and sodium chloride were purchase from Sigma-Aldrich (USA). Caelyx® was kindly provided by the Pharmacy of the University Hospital of Coimbra (Portugal).

### Cell culture

4T1 [19, 26] (ATCC^®^ CRL-2539^™^, USA) mycoplasma-free cells were cultured in RPMI-1640 (Sigma-Aldrich, USA) supplemented with 10% (v/v) heat-inactivated Fetal Bovine Serum (Invitrogen, USA), 100 U/ml penicilin, 100 μg/ml streptomycin (Lonza, Switzerland) and maintained at 37°C in a 5% CO_2_ atmosphere.

### *In vivo* experiments

Cell suspensions were prepared at 5 x 10^3^, 2 x 10^4^, 5 x 10^5^ and 10 x 10^6^ cells/mL in phosphate-buffered solution, and maintained at room temperature. Five to six weeks old Balb/c female mice (Balb/cAnNCrl) were orthotopically inoculated within 40 min after preparation of cell suspension, in the fourth inguinal mammary fat pad (100 μL/mouse).

For the cell titration, mice were assigned to different groups according to the number of 4T1 cells to be injected: 500, 2000, 5 x 10^4^ and 1 x 10^6^. Tumor volume was measured with a caliper every other day, and determined based on the equation π/6(*a* x *b*^2^), where *a* is the largest tumor diameter and *b* is the smallest [27]. Tumors were allowed to grow between 100–200 mm^3^ or > 250 mm^3^, after which animals were euthanized for necropsy and organs harvested for histological analysis.

For the therapeutic study, mice were orthotopically inoculated with 500 4T1 cancer cells *per* mouse. Mice bearing 100–150 mm^3^ tumors were intravenously treated with Caelyx® at 5 mg doxorubicin/kg body weight/week, for five weeks. An additional group was injected with saline.

The development of clinical signs of distress caused by the metastatic disease and body weight losses higher than 20% were not consented and were reason for animal euthanasia. Upon necropsy, the organs were removed, weighed and processed for histological analysis.

All animal experiments were conducted according to human standards of animal care (2010/63/EU directive and Portuguese Act 113/2013, for the use of experimental animals).

### Relative tumor volume, metastatic incidence and metastatic burden

Mean relative tumor volume was expressed as the percentage of the ratio between the tumor volume in each time point and at the beginning of the treatment. Metastatic incidence was determined by the ratio between the number of mice that developed metastases, upon histological confirmation, and the total number of mice assessed. The weight of the lungs was used as a measure of the metastatic burden in this organ, as extensively reported by others [28–31] and confirmed by us, upon comparing non-tumor- or tumor-bearing mice, with or without treatment with Caelyx® (S1 Fig). Relative weight of the organs was expressed as percentage of body weight at the time of death.

### Tumor growth curves

Exponential and Gompertz mathematical models [32] were fit to the mean tumor volume data (overtime) of all mice using non-linear regression, and goodness-of-fit of the models was compared through the Akaike’s Information Criteria (AIC) values and the extra sum-of-squares F test. Specific growth rate (SGR) and doubling time (DT) were determined and given in the output of the fitting analysis.

### Histological analysis of primary tumors and metastases

Primary tumors and organs were kept on fixative solution (Tissue-Tek® Xpress® Molecular Fixative, Sakura) for 24 h, after which tissues were paraffin embedded, sectioned onto slides (4 μm) and stained with hematoxylin/eosin (H&E) or mouse anti-CD31 monoclonal antibody (clone JC70, pre-diluted ref. n° 760–4378, Ventana Medical Systems, Inc., USA) using the BenchMark ULTRA IHC/ISH staining module (Ventana Medical Systems, Inc., USA). Mean vascular density was the mean of CD31 stained blood vessels counted in 4 different fields (400x) of a primary tumor section.

H&E stained sections were visualized in a Axioskop 2 Plus microscope (Zeiss, Germany) for the histological evaluation of metastases and primary tumors invasion of surrounding tissues. Viable rim area was determined in the entire section by excluding necrotic areas, and represented as a ratio of the total area of that section, analyzed with the Fiji software (Life-Line version, 2014 November 25, NIH, USA).

### Statistical analysis

Data were analyzed using unpaired nonparametric one-way *ANOVA*, followed by Dunn’s multiple comparisons test, except when only two groups were compared, in which case unpaired nonparametric Mann-Whitney test was applied. Viable rim area and vessel density for different tumor sizes were analyzed using unmatched two-way *ANOVA* with Tukey’s multiple comparisons test. Log-rank test was applied for the survival curves and metastatic incidence in the therapeutic study was analyzed with a two-tailed chi-square test. All the analyses were performed with a 95% confidence interval.

## Results

### Metastatic pattern and efficiency

The 4T1 metastatic breast carcinoma model is amply used. However, a large number of cells are often implanted in mice [15, 19–24] and require primary tumor removal to extend the disease time course, besides presenting a low metastatic efficiency. Herein, we assessed the effect of inoculated 4T1 cell density on the metastatic efficiency, without removal of the primary tumor. Immunocompetent Balb/c female mice were orthotopically injected with four cell densities, ranging from 500 to 1 x 10^6^ cells, and tumor incidence, time for tumor onset, and metastatic efficiency were evaluated.

No obvious correlation was detected between tumor incidence and the inoculated 4T1 cell density (Table 1). The percentage of mice that developed breast carcinomas varied from 85% to 92%, in animals implanted with 5 x 10^4^ and 1x10^6^ cells, respectively. However, the mean time for tumor onset was significantly different between the groups inoculated. Palpable tumors were detected at 17.5 and 16.5 d *post* injection of 500 and 2000 cancer cells, respectively, whereas this latency time drastically decreased when 5 x 10^4^ (7.6 d, *p* < 0.01) or 1 x 10^6^ (3.6 d, *p* < 0.0001) 4T1 cells were implanted (Table 1).

**Table 1:** Tumor growth and metastases in 4T1 breast carcinoma-bearing mice.

No. 4T1 cells inoculated	No. of mice with tumors/total mice (%)	Mean tumor onset (days until palpable tumors ± SEM)	Time from inoculation to euthanasia (days ± SEM)	Mean tumor volume at euthanasia (mm^3^ ± SEM)	No. of mice with detectable lung metastases/no. of mice evaluated (%)
1x10^6^	11/12 (92%)	3.6 ± 2.54 ^a^	18.4 ± 1.26 ^a^	229.5 ± 45.82	5/11 (45%)
5x10^4^	11/13 (85%)	7.6 ± 0.84 ^b^	24.9 ± 2.28 ^a^	210.6 ± 31.44	5/11 (45%)
2000	13/15 (87%)	16.5 ± 1.05	35.4 ± 1.65	285.9 ± 44.35	7/9 (78%)
500	13/15 (87%)	17.5 ± 0.91	37.0 ± 2.08	318.1 ± 34.05	7/7 (100%) ^c^

^a^ *p* < 0.0001.

^b^ *p* < 0.01 relative to 500 and 2000 cell densities (nonparametric one-way *ANOVA* with Dunn’s multiple comparisons test).

^c^ *p* = 0.0167, relative to 5x10^4^ and 1x10^6^ cell densities (two-tailed chi-square test).

The time course of primary tumor growth might have implications on its metastatic efficiency. In fact, the number of mice with detectable lung metastases significantly increased in those groups with the longest tumor onset, achieving 100% efficacy on the group where only 500 cells were inoculated (Table 1). In contrast, only 45% of mice developed lung metastases (or metastatic nodules in other tissues), for cell densities superior to 5 x 10^4^. As the endpoint of this experiment was a similar primary tumor burden across the four groups (*p* = 0.1938), the time elapsed from cell inoculation to animals sacrifice was significantly reduced in the groups injected with 5 x 10^4^ and 1 x 10^6^ 4T1 cells (Table 1). In these experimental conditions, the metastatic process was more efficient when lower numbers of 4T1 cells were inoculated. Nonetheless, the observed differences in the metastatic efficiency will likely be attenuated if the animals inoculated with higher cell densities were allowed to live longer (a condition that would be associated with higher tumor burdens).

**Fig 1:**
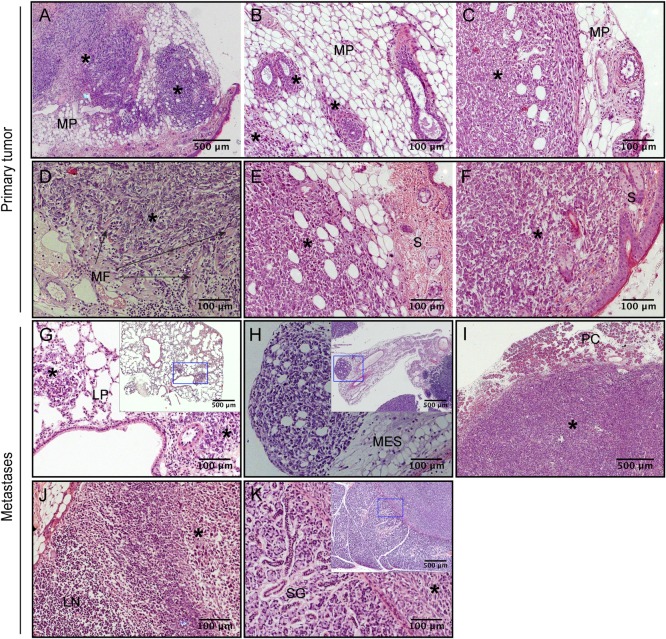
Representative sections from orthotopic 4T1 tumors and nodular metastatic deposits. Tumor cells invading the surrounding mammary parenchyma (A—C), muscle fibers (D) and adjoining skin (E–F) show the highly invasive capacity of 4T1 breast tumors. Examples of metastatic lesions were observed in the lungs (G), mesentery (H), pancreas (I) lymph nodes (J), or salivary gland (K). MP, mammary parenchyma; MF, muscle fibers; S, skin; LP, lung parenchyma; LN, lymph node; MES, mesentery; PC, pancreas; SG, salivary gland; * indicates tumor areas. All images present original magnification x200, except upper left and inset images, x50.

Fig 1 shows representative images of 4T1 breast tumors and metastatic lesions in several tissues. The highly invasive nature of these tumors was confirmed by their capacity to invade neighboring mammary parenchyma (Fig 1A–1C), muscle fibers (Fig 1D) and adjacent skin (Fig 1E and 1F). Macrometastatic lesions in several organs and tissues were also observed (Fig 1G–1K). In these experiments, 4T1 cells have colonized mainly the lungs (Fig 1G), as previously described [19, 33], while macrometastases in the liver were rarely observed. As important, macrometastases in the brain were not identified. Metastases in tissues such as the mesentery (Fig 1H) and pancreas (Fig 1I) were often observed, and occasionally in lymphatic nodes (Fig 1J). Less frequently, other tissues, like the salivary gland (Fig 1K), were affected. However, the extent of pulmonary and visceral lesions was considerably variable amongst individuals. Different metastatic patterns were observed in mice, particularly on the lowest cell densities: either small lesions extensively spread, or scarce but larger metastatic nodules. Therefore, it was difficult to establish the metastatic burden based on the number or volume of the lesions. Moreover, it was difficult to perform weight estimates in certain tissues, such as the mesentery.

### Dynamics of 4T1 tumor growth

**Fig 2:**
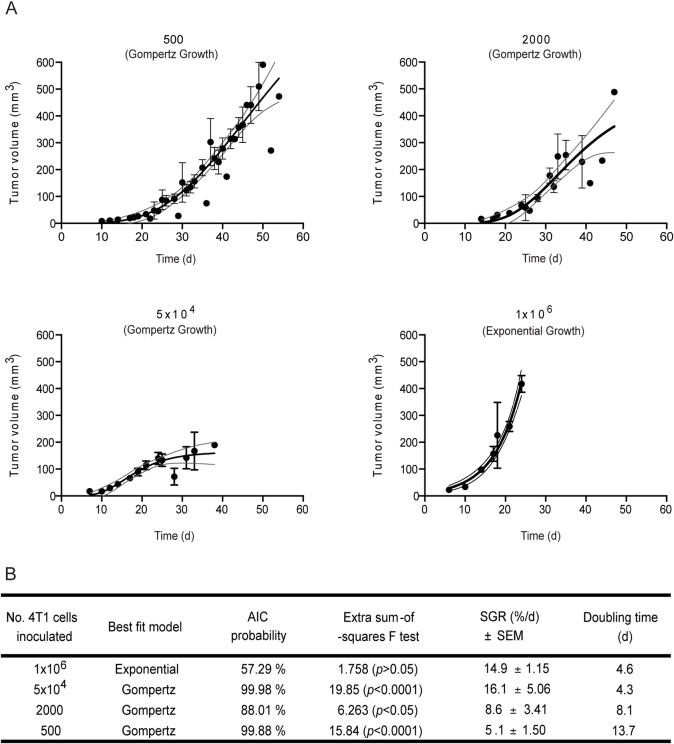
Fitting mathematical growth models to tumor experimental data as a function of inoculated cell density. Exponential and Gompertz models were fitted to the population’s tumor growth curves (A) and compared using the Akaike’s information criteria (AIC) and the extra sum-of-squares F test (B). Specific growth rates (SGR) and doubling times of each group were determined from the mathematical equations of the best fit (B). Dark symbols represent experimental mean tumor volumes. The solid line represents the best fit for each group with a 95% confidence interval, light lines.

Dynamics of tumor growth were analyzed by two mathematical models commonly used to describe tumor growth: the Exponential and Gompertz models [34]. The first is a simplistic model that assumes that the number of cancer cells doubles during cell cycle, resulting in exponential growth of solid tumors. However, tumor growth involves other biological processes, such as regulation of proliferation, stromal recruitment, escape from immunesurveillance and angiogenesis, thereby being usually explained by the Gompertz model, which considers growth rate decay as tumors become larger [35, 36]. Tumor growth curves fitted to the experimental mean tumor volumes over time are presented in Fig 2A. The model providing the best fit was chosen based on Akaike’s information criteria and extra sum-of-squares F test (Fig 2B) analysis [37, 38].

Tumor growth showed a Gompertz behavior for all groups, except for those generated from inoculation of 1x10^6^ cells, where exponential growth was prevalent (Fig 2). Notwithstanding, Gompertzian tumor growth was diverse among the different groups. It was evident that tumors generated from 5 x 10^4^ cells attained the decay phase much earlier than the tumors resulting from the inoculation of lower cell densities (Fig 2A).

Doubling time and specific growth rate, two parameters usually used to quantify and characterize neoplastic growth, were also determined. One million and 5 x 10^4^ cell density groups averaged growth rates of 14.9% and 16.1% *per* day, with doubling times of 4.6 and 4.3 days, respectively (Fig 2B).

### 4T1 tumors viable rim area and vasculature

**Fig 3:**
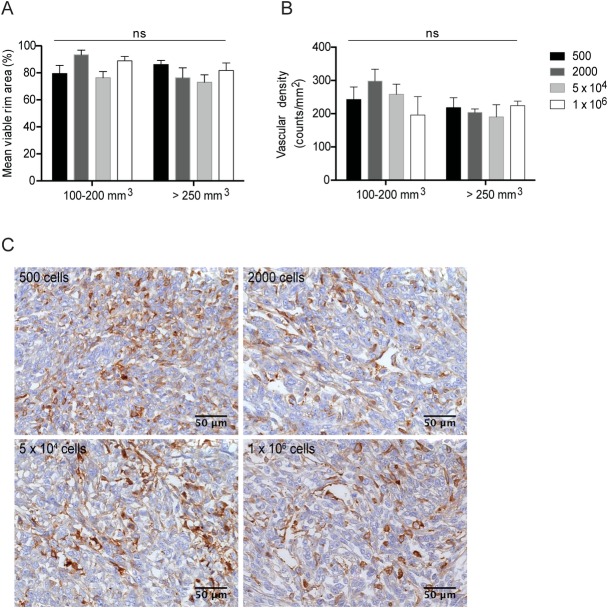
Effect of cell density and tumor mean volume on viable rim area and vascular density. Quantitative analysis of viable rim area (A) and vascular density (B) was assessed in tumors with mean volumes of 100–200 mm^**3**^ and > 250 mm^**3**^, based on H&E or CD31 immunostaining in carcinoma sections derived from 500, 2000, 5 x 10^**4**^ and 1 x 10^**6**^ cancer cells, original magnification x400 (C). Data represent the mean ± SEM of 3–6 independent sections. ns, *p* > 0.05 two-way *ANOVA* with Tukey’s multiple comparisons test.

Tumor necrosis is thought to result from rapidly proliferating cancer cells outpacing their blood supply in certain tumor regions [39, 40]. As necrotic cells release pro-inflammatory factors into the tumor microenvironment, which are known to promote tumor growth and dissemination [39, 41], it was further questioned whether the altered dynamics would affect the viable rim area and vascular density of primary tumors, and whether it would relate to their metastatic efficiency. Sections from tumors of all groups were stained either with H&E, to determine the viable rim area, or with CD31, to assess vascular density. Additionally, the analysis accounted for differences on tumor volumes, distinguishing between smaller (100–200 mm^3^) and larger (> 250 mm^3^) tumors, within each group of mice. Both the viable rim area and vascular density were independent from tumor volume and cell density inoculated (Fig 3A and 3B, respectively). Representative images of tumor sections stained with CD31 (Fig 3C) confirmed the high vascularized nature of these tumors, regardless the number of inoculated cells they were generated from. These tumors already entailed a good vascular network at volumes between 100–150 mm^3^.

Overall, neither the viable rim area nor the vascular density of tumors originating from the different cell densities correlated with their respective metastatic efficiency. Nevertheless, the inoculation of 500 4T1 cancer cells provided the best metastatic efficiency, possibly due to lower specific growth rates (or extended doubling times), yielding an optimal model to study metastatic breast cancer.

### Validation of the established conditions for the 4T1 metastatic breast cancer mouse model

In order to validate the previously characterized 4T1 metastatic breast carcinoma mouse model, a therapeutic study was conducted with pegylated liposomal doxorubicin (Caelyx®), a cytotoxic agent used in the clinical setting of metastatic breast cancer.

**Fig 4:**
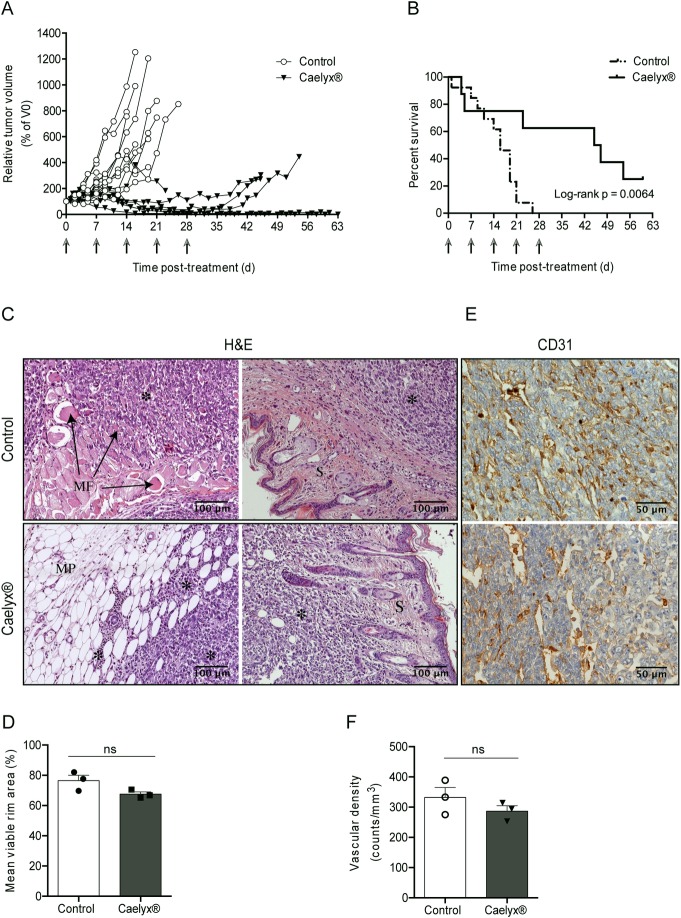
Therapeutic activity of Caelyx® in 4T1 breast tumor-bearing mice. Balb/c mice implanted with 500 4T1 cancer cells in the mammary fat pad were treated weekly with Caelyx®, at 5 mg doxorubicin/kg body weight for 5 weeks (indicated by grey arrows). An additional control group included non-treated mice (injected with saline). Individual tumor growth curves (A) and survival curves (B) are illustrated. H&E staining, original magnification x200 (C), quantification of viable rim area (D), CD31 immunostaining, original magnification x400 (E), and quantification of vascular density (F), on representative sections from 4T1 primary tumors, following treatment either with Caelyx® or saline. MF, muscle fibers; S, skin; MP, mammary parenchyma; * indicates tumor areas. Data represents individual relative tumor volumes and median survival curves of saline- (n = 14) and Caelyx®-treated (n = 8) mice. Viable rim area and vascular density represent mean ± SEM of 3 independent sections. ns, *p* > 0.05 two-tailed nonparametric Mann-Whitney test.

Upon inoculation of 500 4T1 cancer cells in the mammary fat pad, Balb/c mice were monitored for body weight and symptoms of distress caused by metastatic disease [42, 43]. In view of the preceding results, therapeutic protocol was initiated when tumors presented an established vascular network (100–150 mm^3^). Mice were weekly treated via the lateral tail vein with Caelyx®, at 5 mg doxorubicin/kg body, for 5 weeks, and a control group was injected with saline. Due to widespread of metastatic disease, not all animals completed this therapeutic regimen. The majority of mice in the control group survived up to the 3^rd^ dose, and only those treated with Caelyx® managed to achieve the 5^th^ administration. A significant reduction of the primary tumor was observed in three Caelyx®-treated mice, although followed by regrowth, as well as two complete remissions (Fig 4A). The latter remained tumor-free for more than 60 days after the last dose and did not present metastatic nodules upon necropsy. Nevertheless, three animals responded poorly to the Caelyx® therapy and died from the disease (Fig 4B). Non-treated mice presented quite a different response, namely in terms of primary tumor growth (*p* < 0.0001 relative to Caelyx-treated mice, Fig 4A) and overall survival (median 16 days versus 46 days in the Caelyx®-treated group, Fig 4B; Log-rank *p* = 0.0064.

Histological analysis of the neoplastic tissues confirmed the vast capacity of 4T1 breast cancer cells to invade muscle, skin and surrounding mammary parenchyma (Fig 4C). Caelyx® did not limit tumoral invasiveness (Fig 4C), nor induced a significant reduction of the viable rim area as compared to non-treated mice (Fig 4D). This effect was consistent with the comparable mean vascular density (Fig 4E and 4F) observed between treatment with Caelyx® (287 ± 17.95 counts/mm^2^) and non-treated mice (332 ± 32.91 counts/mm^2^).

Secondary metastatic lesions in several organs/tissues were also observed in all animals, with the exception of the two mice that presented a complete response to Caelyx® (Table 2).

**Table 2:** Incidence of metastatic lesions in 4T1 breast carcinoma-bearing mice.

	No. of mice with metastatic lesions /total no. of mice analyzed (%)				
	Lungs	Liver	Pancreas	Mesentery	Diaphragm
Control	13/13 (100%)	2/13 (15%)	9/13 (69%)	10/13 (77%)	6/13 (46%)
Caelyx®	6/8 (75%)^**a**^	0/8 (0%)	4/8 (50%)	3/8 (38%)^**b**^	2/8 (25%)

^a^, *p* = 0.0581.

^b^, *p* = 0.0708 (two-tailed chi-square test analysis).

**Fig 5:**
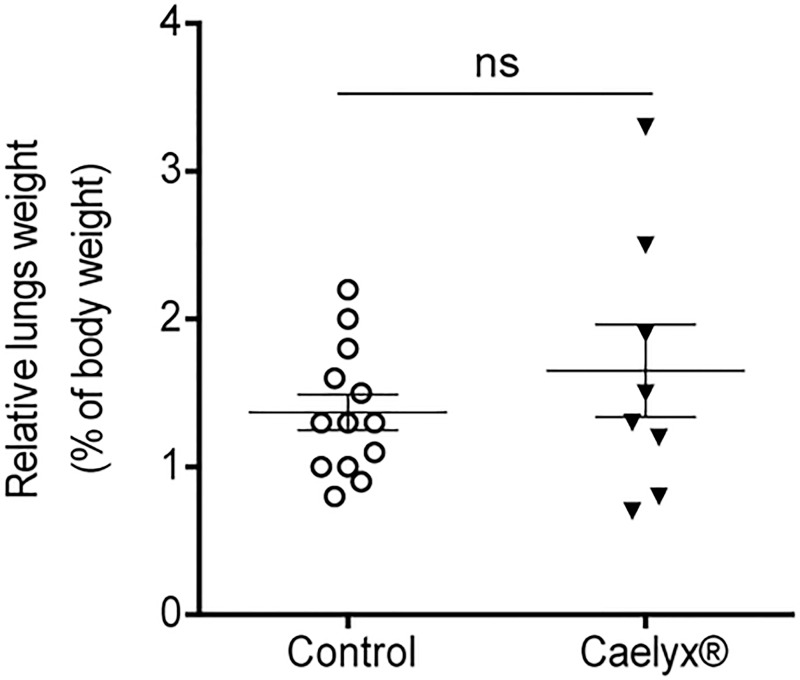
Metastatic burden in the lungs following treatment with Caelyx®. Metastatic burden was determined upon organ weight for individual mice treated either with Caelyx® or saline, and further normalized for the whole body weight. Data represent individual (dots) or mean **±** SEM of control (n = 13) and Caelyx® (n = 6) mice. ns, *p* > 0.05 two-tailed nonparametric Mann-Whitney test.

One hundred percent of non-treated animals presented lung metastases. The incidence decreased in the Caelyx® cohort (75%) owing to disease remission in two mice. Noteworthy, the results also pointed to a reduction of mesenteric nodules in mice under Caelyx® therapy compared to non-treated mice (38% *versus* 77%, *p* = 0.0708), with the contribution of the two disease-free mice at the end of the experiment (Table 2). Despite lung metastatic burden of non-treated animals (1.37 ± 0.12) was comparable to the one of Caelyx® group (1.65 ± 0.31), Fig 5, the latter presented the longest survival rate (Fig 4B). Notwithstanding the significant effect of Caelyx® on primary tumor growth inhibition, the extended survival time of these animals enabled a sufficient time frame for metastatic development, already *in site* by the time of treatment initiation.

**Fig 6:**
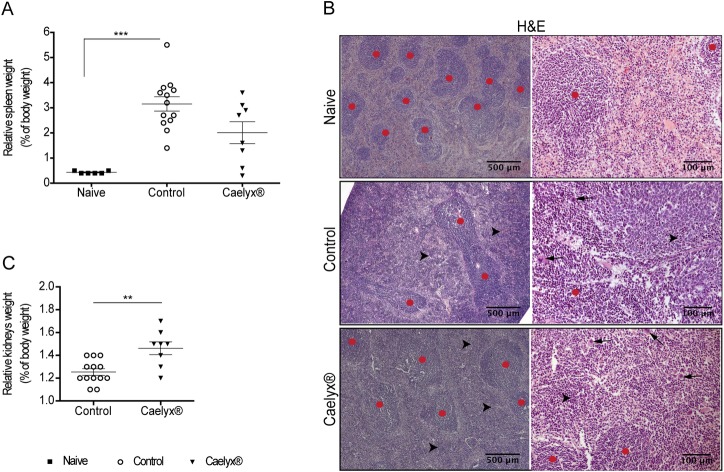
Effect of Caelyx® in the spleen and kidneys of mice bearing 4T1 tumors. Relative weight (A) and representative H&E images, original magnification x50 (left) or x200 (right) (B) of spleens from naive mice and mice treated with Caelyx® or saline (control). Images illustrate the red pulp markedly expanded by numerous hematopoietic cells (black arrowheads), including megakaryocytes (black arrows), and myeloid precursor cells, particularly in the controls. Red circles represent white pulp areas with lymphoid nodules. The relative weight of kidneys (C) from Caelyx®- or saline-treated control mice was also analyzed. ***, *p* < 0.001, nonparametric one-way *ANOVA* with Dunn’s multiple comparisons test; **, *p* < 0.01, two-tailed nonparametric Mann-Whitney test.

Splenomegaly was confirmed by visual examination and relative organ weight quantification, in all mice bearing 4T1 tumors compared to naïve animals (Fig 6A), in agreement with data from other studies [33, 44]. Interestingly, treatments with Caelyx® resulted in a slight decrease of relative spleen weight, in comparison with non-treated mice (Fig 6A). Histological examination of the spleens revealed hyperplasia of the red pulp with a concomitant reduction of the white pulp, with particular emphasis in non-treated mice (Fig 6B), and consistent with extramedullary hematopoiesis.

Nonetheless, some degree of toxicity might have occurred given the increase of mean relative kidneys weight in Caelyx®-treated animals (*p* values = 0.0048), relative to non-treated mice (Fig 6C). Symptoms of palmar-plantar erythrodysesthesia were not registered in mice treated with Caelyx®, in contrast with previous reports [45], possibly due to a lower dose of doxorubicin used herein.

## Discussion

The orthotopic inoculation of cancer cells is extensively used in breast cancer research. The predictive utility of these tumor models relies on their capacity to reproduce human malignancy, and should allow the analysis of human primary tumor growth, including invasion of surrounding tissue, interactions of tumor cells with their stromal components, and metastatic progression [6, 8]. Breast cancer originates from genetic and epigenetic transformations in a single cell, progressing to clonal expansion and selection and, ultimately, to metastatic disease [46]. Animal models of breast cancer have provided some understanding of these features, enabling the proposal of new treatments [47]. However, primary breast tumors often originate from the inoculation of a large number of cancer cells, which is associated with a high growth rate, failing to efficiently generate metastases or leading to their surgically resection to prolong survival [6]. Therefore, this strategy clearly deviates from the natural history of the disease, resulting in neoplastic tissues that, from our experience, do not resemble the human malignancy. Likewise, the 4T1 breast carcinoma mouse model is frequently implemented upon inoculation of a large number of 4T1 cancer cells in the mammary fat pad of mice, ranging from 1 x 10^4^ to more than 1x10^6^ cells [15, 19–25]. However, in the present work it has been demonstrated that the inoculation of such high cell densities compromised the development of an efficient and reproducible model of spontaneous metastases.

It was demonstrated that 4T1 tumors grew orthotopically when as few as 500 cancer cells were inoculated, being tumor incidence comparable for cell densities ranging from 500 to 1x10^6^ (87% and 92%, respectively). However, inoculated cell density influenced significantly the latency period for first palpation and metastatic efficiency (Table 1). The indirect correlation between the number of inoculated cancer cells and the time required to proliferate and generate a larger tumor mass, was in contrast with the effect on the metastatic efficiency. Only 45% of mice inoculated with 1x10^6^ cells presented macroscopic secondary lesions, which is in contrast with the 100% metastatic efficiency of the group inoculated with 500 cells (Table 1). Interestingly, Bailey-Down *et al*. [48] also showed that reducing the number of implanted 4T1 cells increased metastatic efficiency. Nevertheless, in animals inoculated with a cell density equal or lower than 1500, the tumor take rate was lower than 50% and macrometastases were barely observed at five weeks post-implantation [48], in contrast with results reported herein. In the work of Bailey-Down *et al*., the best compromise between tumor take rate (over 90%) and metastatic efficiency (67% in the lungs) was achieved upon inoculation of 7500 4T1 cells. It is well known that the shedding of cancer cells into the bloodstream is an early event in the process of tumorigenesis [49, 50]. Therefore, one could assume that tumors originated from different cell densities would enable similar metastatic potential. However, in the groups inoculated with the highest cell densities, the time frame between tumor onset and animal euthanasia was strongly shortened by the high tumor burden and subsequent need of primary tumor resection. This short time frame was not sufficient for the progression of seeded cells into macrometastases. Therefore, the present results clearly demonstrated that the inoculation of low cell densities surpass the need of primary tumor resection, thus becoming a key methodological aspect to the reproducible development of macrometastases in the 4T1 mouse model.

The results herein presented also highlighted the impact of cancer cell density on the dynamics of the primary tumor development, as reflected by the different growth curves, and specific growth rates and doubling times (Fig 2). In fact, tumors originated from 5x10^4^ and 1x10^6^ cells grew at a rate approximately 2 and 3 times faster than those from 2000 and 500 cell densities, respectively, being in line with the corresponding shortened time for tumor onset (Table 1). Two conceptual theories, the Exponential [34] and Gompertz [34, 35] growth models, have been used to describe the tumor dynamics. The first one states that primary tumor expansion is purely the result of cell division, resulting in exponential growth of solid tumors, while the second incorporates different growth rates, depending on the stage of tumor development [35]. The cancer cell inoculation titration herein performed was better described by the Gompertz model, but only to a certain extent, as tumors generated from 1x10^6^ cells followed an exponential growth. An old concept to explain the Gompertz model is the insufficient nutrient supply to large solid tumors, which impairs their unlimited expansion [51], thus justifying the decrease of growth rate upon reaching a certain mean volume. Yet, evidence of large tumors (1–2 cm^3^) with an adequate vascular network [52] has challenged this theory, and is in agreement with the results obtained herein showing similar mean vascular densities (Fig 3B) and viable rim areas (Fig 3A), regardless tumor burden and inoculated cell densities. More recently, the idea of self-seeding [36] as a multidirectional process, whereby cancer cells seed secondary sites and re-infiltrate the primary *foci*, has been validated in several experimental models, including the 4T1 breast cancer model [53]. Self-seeding selects for highly aggressive fractions of circulating tumor cells based on their movement and survival in the bloodstream. The primary tumor homing of this population of cells was shown to further induce angiogenesis and invasion, in addition to the breeding of metastatic progenies in a compatible soil [53]. This process is thought to contribute for the Gompertzian expansion of tumors [36] and might be also contributing for the high metastatic efficiency observed for the lowest 4T1 cell densities.

The higher metastatic efficiency of the 500, 2000 and 5x10^4^ cell density groups may result from other factors as well. 4T1 cancer cells were shown to play an active role in the production and release of a variety of chemokines [44, 54–57] that stimulate hematopoiesis, enabling the recruitment and activation of inflammatory cells to the primary tumors and metastatic lesions, and modulation of the endothelial function [33, 44, 58–60]. Among the inflammatory cells infiltrating the 4T1 tumors, a CD11b^+^ myeloid population with the F4/80^+^CD11c^+^ Gr-1^+^ phenotype was the most prominent, increasing over time, in parallel with tumor growth, while lymphoid cells decreased [44]. Although we did not look into systemic and microenvironment local changes, the occurrence of splenomegaly was observed, suggesting that a leukemoid reaction took place (Fig 6A and 6B). duPré and Hunter have previously correlated splenomegaly in the 4T1 tumor-bearing mice with a prominent increase in immature splenic granulocytes, along with a reversal of the splenic lymphocyte:granulocyte ratio, with decreased percentages of B- and T-cells [56]. Waight et al. further confirmed a marked increase in splenic CD11b+Gr-1+ cells of 4T1 tumor-bearing mice in response to granulocyte colony-stimulating factor (G-CSF) [61]. These events stimulated primary *foci* progression and, ultimately, metastasization by promoting cancer cell invasion, dissemination, seeding and growth into metastatic lesions [56, 61–63]. Nevertheless, all these processes require a minimum time frame to occur. As such, the time frame requested for the expansion of seeded cells into macrometastases could explain the lower metastatic efficiency of 5x10^4^ cell inoculated group, as compared to the 500 and 2000 counterparts, despite all follow the Gompertz growth model.

In the therapeutic study, no physical signs of toxicity were attained by treatment with Caelyx®, albeit kidneys enlargement was observed in these mice (Fig 6C). Although Caelyx® was not proficient in fully preventing metastatic disease (Table 2), it enabled a significant therapeutic response (Fig 4A and 4B). The reduction of primary tumor burden promoted by Caelyx® was a major contributor for the observed median overall survival, and reduced the metastatic incidence in the mesentery (Table 2). The antitumor efficacy of Caelyx® in animal models is the result of the enhanced permeability and retention (EPR) effect, to which the high circulation half-life in the blood and subsequent passive extravasation at the level of the leaky tumor blood vessels have a major contribution [64–66]. Therefore, the presence of an established vasculature is essential for the therapeutic activity of Caelyx®. The absence of a vascular network or the existence of a poorly developed vasculature in lung metastases, as compared to the primary tumor, may justify the high metastatic burden despite treatment with Caelyx®. Nevertheless, and since colonization of the visceral organs by cancer cells in the 4T1 mammary carcinoma model was described to occur later than the lungs [19, 33, 48], it is possible that the liposomal chemotherapy tested herein, limited further dissemination/colonization of tissues of the peritoneal cavity by 4T1 cells, accounting for a lower incidence of metastasis in the mesentery. Extramedullary hematopoiesis has been shown to play a crucial role in the spontaneous metastization in the 4T1 mouse model [44, 56], by contributing to a receptive microenvironment for circulating tumor cells arrest, survival and proliferation in the lungs [44, 67, 68]. Therefore, the therapeutic activity of Caelyx® could be partially exerted by restricting splenic hematopoiesis, as reflected in a reduced splenomegaly, relative to non-treated mice (Fig 6A and 6B). Likewise, it has been demonstrated in mouse models of glicosarcoma, neuroblastoma and lung cancer [69, 70] that Caelyx® exerted its antitumoral activity in part by the ability to elicit an antivascular effect. This effect was not observed herein with the 4T1 model, possibly due to the aggressiveness of the cancer model used herein and/or dose scheduling. The low extent of the antivascular effect might support the similar tumor viable rim area between mice treated with Caelyx® and non-treated mice (Fig 4D). Additionally, non-tumoral vessels from surrounding tissues are also expected to provide nutrient and oxygen supplies to cancer cells [71, 72]. These factors likely contributed to the maintenance of tumor viable areas that fuels its expansion and invasion (Fig 4C), and could be responsible for the tumor regrowth observed in some Caelyx®-treated mice.

## Conclusions

Notwithstanding the 4T1 mouse model being widely used to study metastatic breast cancer, the low metastatic efficacy is barely mentioned in the literature. Herein, it was demonstrated that reducing the number of 4T1 cancer cells implanted orthotopically, to a number as low as 500 cells, resulted both in a higher metastatic efficiency and primary tumor take rate, significantly affecting the dynamics of tumor growth. This becomes a key methodological aspect towards the reproducible development of macrometastases in the 4T1 mouse model, as validated in the therapeutic experiment. Extending the time length of tumor development will enable longitudinal studies to follow all the steps of cancer cell dissemination in the same individual, as well as a better assessment of anti-metastatic therapies.

## Supporting Information

S1 Fig Metastatic burden in the lungs of non-tumor- or tumor-bearing mice, with or without treatment with Caelyx®. Metastatic burden (A) was determined upon organ weight for individual mice, either naïve, non-treated (control) or treated with Caelyx®. Representative images of the lungs (B) from non-treated (control) or Caelyx®-treated mice and their correspondent weight. Relative lungs weight, in parentheses, was determined upon normalization for the whole body weight. Data represented individual (dots) or mean ± SEM of naïve (n = 6), control (n = 13) and Caelyx® (n = 8) mice. **, *p* < 0.01 non-parametric one-way *ANOVA* with Dunn’s multiple comparisons test.
(PDF)
