# Supplementary material for: Inoculated Cell Density as a Determinant Factor of the Growth Dynamics and Metastatic Efficiency of a Breast Cancer Murine Model
Source: PLoS One. 2016 Nov 7;11(11):e0165817. doi: 10.1371/journal.pone.0165817 (PMC5098815; doi:10.1371/journal.pone.0165817)
Supplement: S1 Fig — Metastatic burden (A) was determined upon organ weight for individual mice, either naïve, non-treated (control) or treated with Caelyx®. Representative images of the lungs (B) from non-treated (control) or Caelyx®-treated mice and their correspondent weight. Relative lungs weight, in parentheses, was determined upon normalization for the whole body weight. Data represented individual (dots) or mean ± SEM of naïve (n = 6), control (n = 13) and Caelyx® (n = 8) mice. **, p < 0.01 non-parametric one-way ANOVA with Dunn’s multiple comparisons test. (PDF) [file pone.0165817.s001.pdf]

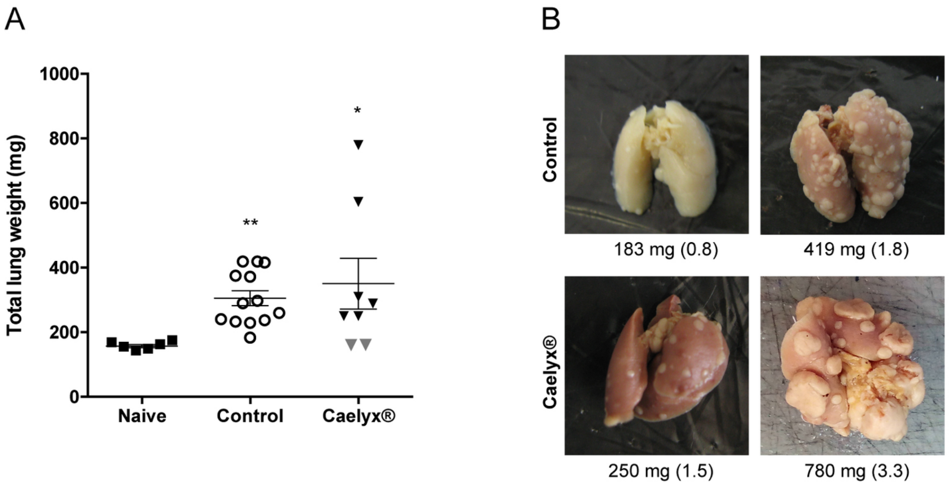

S1 Fig: Metastatic burden in the lungs of non-tumor- or tumor-bearing mice, with or without treatment with Caelyx®.

Metastatic burden (A) was determined upon organ weight for individual mice, either naïve, non-treated (control) or treated with Caelyx®. Representative images of the lungs (B) from non-treated (control) or Caelyx®-treated mice and their correspondent weight. Relative lungs weight, in parentheses, was determined upon normalization for the whole body weight. Data represented individual (dots) or mean ± SEM of naïve (n=6), control (n=13) and Caelyx® (n=8) mice. **, $p < 0.01$ non-parametric one-way *ANOVA* with Dunn's multiple comparisons test.

S1A Fig illustrates the significant difference in the mean total weight of the lungs between naïve mice (157.0 ± 4.98 mg) and non-treated (350.4 ± 23.30 mg, p=0.0023) or Caelyx®-treated mice (350.5 ± 78.67 mg, p=0.0131), data that reported to the therapeutic experiment presented in our manuscript. Moreover, the lungs of the two mice without metastases, in the Caelyx®-treated group, as confirmed by histological examination, weighed 160 and 161 g, respectively, which were within the range of those of naïve mice (141 – 173 g). Lastly, in S1B Fig images of the lungs from non-treated (control) or Caelyx®-treated mice, and their corresponding total weights, are presented, further illustrating the direct correlation between lung weight and the metastatic load in the organ.
